# scHiCDiff: detecting differential chromatin interactions in single-cell Hi-C data

**DOI:** 10.1093/bioinformatics/btad625

**Published:** 2023-10-17

**Authors:** Huiling Liu, Wenxiu Ma

**Affiliations:** Department of Statistics, University of California Riverside, Riverside, CA 92521, United States; Department of Statistics, University of California Riverside, Riverside, CA 92521, United States

## Abstract

**Summary:**

Here, we presented the scHiCDiff software tool that provides both nonparametric tests and parametirc models to detect differential chromatin interactions (DCIs) from single-cell Hi-C data. We thoroughly evaluated the scHiCDiff methods on both simulated and real data. Our results demonstrated that scHiCDiff, especially the zero-inflated negative binomial model option, can effectively detect reliable and consistent single-cell DCIs between two conditions, thereby facilitating the study of cell type-specific variations of chromatin structures at the single-cell level.

**Availability and implementation:**

scHiCDiff is implemented in R and freely available at GitHub (https://github.com/wmalab/scHiCDiff).

## 1 Introduction

The increasing availability of single-cell Hi-C (scHi-C) data promises exciting research opportunities to explore cell type-specific chromatin structures at the single-cell level. However, computational and statistical methods for scHi-C data analysis are at a very early stage. Currently, existing scHi-C methods mainly focus on data normalization (Liu and Wang 2018, Zheng *et al.* 2022) and similarity measures among single cells (Liu *et al.* 2018), while few pay attention to chromatin structure variations between different conditions (Yu *et al.* 2021).

Comparative and differential analysis of scHi-C datasets can facilitate the identification of changes in chromosomal structures, especially chromatin interactions, between different conditions, and thereby shed light on the principles of chromatin structure organization. However, computational methods for detecting differential chromatin interactions (DCIs) in scHi-C data are not yet available. Although several methods have been developed to detect DCIs in bulk Hi-C data (Lun and Smyth 2015, Stansfield and Dozmorov 2017, Djekidel *et al.* 2018), such methods cannot be directly applied to scHi-C, due to the unique features of sparsity and heterogeneity of the single cell data.

Similar to scHi-C data, single-cell RNA-seq (scRNA-seq) data also faced high heterogeneity and excessive zero problems. Recently, several methods have been proposed to detect differentially expressed genes (DEGs) in scRNA-seq data. For instance, D3E (Delmans and Hemberg 2016) used nonparametric Cramér–von Mises (CVM) and Kolmogorov–Smirnov (KS) tests or parametric Poisson-Beta model to compare the distributions of expression values of each gene to identify DEGs. Later, DEsingle (Miao *et al.* 2018) utilized a zero-inflated Negative Binomial (ZINB) regression model to estimate the Negative Binomial (NB) parameters, mean and dispersion, as well as the proportion of the real and drop-out zeros in the observed scRNA-seq data.

Inspired by these scRNA-seq models, we designed and implemented a novel statistical software tool named scHiCDiff, which applied two nonparametric tests (KS and CVM) and two parametric models (NB and ZINB) to distinguish the bin pairs showing significant changes in contact frequencies between two groups of scHi-C data. Specifically, the ZINB regression model accounted for the properties of sparsity and heterogeneity in scHi-C data and performed a rigorous statistical test (likelihood ratio Chi-square test) to detect DCIs at the single-cell level. We demonstrated, through simulation studies and real data analysis, that scHiCDiff can effectively and accurately identify single-cell DCIs between two conditions.

## 2 Materials and methods

**Figure 1.**
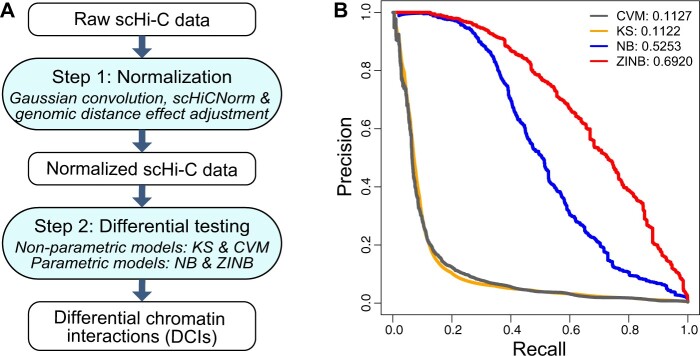
(A) The workflow of scHiCDiff. (B) Performance of scHiCDiff methods on simulated data (fold change = 5, sample size = 50, and resolution = 200 kb).

As illustrated in Fig. 1A, scHiCDiff detects DCIs in two steps: normalization and differential testing. In the normalization step, each scHi-C data is first imputed by a Gaussian convolution filter to tackle the sparsity issue, then processed by scHiCNorm (Liu and Wang 2018) with the Negative Binomial Hurdle option to remove systematic biases, and finally normalized for the cell-specific genomic distance effect (Supplementary Methods; Supplementary Fig. 2).

In the differential testing step, scHiCDiff quantifies and compares distributions of each interaction bin pair between two conditions using either nonparametric approaches (KS or CVM test) or parametric methods (NB or ZINB model) (Supplementary Methods). Specifically, the ZINB model is designed to account for the extreme sparsity of scHi-C data (Supplementary Fig. 1). Here, the task of detecting DCIs between two conditions is equivalent to testing the heterogeneity of two populations. The null hypothesis is that the two groups of single cells are from the same population, hence the contact counts at each bin pair are drawn from the same distribution. Consequently, the hypothesis testing is conducted through KS or CVM test for the nonparametric methods, or via the likelihood ratio Chi-square test for the parametric methods. The detailed description of the four scHiCDiff methods are available in Supplementary Methods.

Note that the differential detection framework accounts for the multiple testing correction using the FDR procedure. The scHiCDiff software is developed in R. The source code is publicly available at https://github.com/wmalab/scHiCDiff.

## 3 Results

### 3.1 scHiCDiff successfully detected DCIs in simulated data

To evaluate the performance of scHiCDiff methods, we first generated two groups of synthetic scHi-C data: one was the control group simulated based on the scHi-C dataset from Nagano *et al.* (2017), and the other was the treatment group with a predefined set of DCIs. Then we compared the performance of the four methods in scHiCDiff on these simulated data, with respect to fold change, resolution, and sample size (Supplementary Methods). Our results demonstrated that all four methods can effectively detect DCIs in scHi-C data (Fig. 1B). Overall, the parametric models (NB and ZINB) outperformed the nonparametric tests (KS and CVM). In addition, the ZINB model was specifically designed for sparse scHi-C data and therefore produced the most accurate results in all simulation settings (Supplementary Fig. 2; Supplementary Note 1).

### 3.2 scHiCDiff effectively revealed cell type-specific DCIs

Next, we applied scHiCDiff on two real scHi-C datasets to identify DCIs between mouse oocyte and zygote cells (Flyamer *et al.* 2017) and between human astrocytes (Astro) and endothelial cells (Endo) (Lee *et al.* 2019). For quality control, we only considered the cells that contained more than 5000 total nondiagonal intra-chromosomal contacts and the contacts within the range of 0–10 Mb for subsequent analyses (Supplementary Methods).

Our results showed that scHiCDiff effectively detected bin pairs that had significant changes in their interactions between different cell types (Supplementary Note 2). In particular, the ZINB model provided consistent and stable DCI detection results and outperformed the other three models (Supplementary Notes 3 and 4).

In addition, we demonstrated that scHiCDiff outperformed the set-difference approach of calculating the difference between significant chromatin interactions in two conditions (Supplementary Note 5). Furthermore, we found that a majority of the ZINB-detected DCIs were supported by DCIs identified by bulk Hi-C methods and were located within differential TAD regions (Supplementary Note 6). Moreover, the genomic bins present in DCIs were associated with significant changes of epigenetic marks between Astro and Endo cells, and Gene Ontology analysis revealed a high enrichment of genes associated with neuron transmitter and synaptic membrane pathways in DCI sites (Supplementary Note 7).

## 4 Conclusion

We developed a novel statistical method scHiCDiff to identify DCIs between two conditions at the single-cell level. In the scHiCDiff framework, we introduced a ZINB regression model to account for the extreme sparsity and heterogeneity in scHi-C data. Through the performance evaluation and differential analysis of simulated and real data, we demonstrated that scHiCDiff provides a rigorous statistical tool for detecting DCIs at the single-cell level, thereby greatly facilitating the comparative analyses of chromatin structures in scHi-C data.

## Supplementary Material

btad625_Supplementary_Data
