# Supplementary material for: scHiCDiff: detecting differential chromatin interactions in single-cell Hi-C data
Source: Bioinformatics. 2023 Oct 17;39(10):btad625. doi: 10.1093/bioinformatics/btad625 (PMC10598576; doi:10.1093/bioinformatics/btad625)
Supplement: btad625_Supplementary_Data [file btad625_supplementary_data.pdf]

Supplementary Information for "scHiCDiff: Detecting Differential Chromatin Interactions in Single-cell Hi-C Data"

Huiling Liu & Wenxiu Ma

Supplementary Information

Supplementary Methods **3**
1 Data imputation and normalization . . . 3
2 Detecting differential chromatin interactions by non-parametric tests . . . 4
2.1 Kolmogorov–Smirnov test . . . 4
2.2 Cramér-von Mises test . . . 4
3 Detecting differential chromatin interactions by parametric models . . . 5
3.1 Negative Binomial model . . . 5
3.2 Zero-inflated Negative Binomial model . . . 7
4 Simulation setting . . . 8
5 Real data pre-processing . . . 9

Supplementary Notes **10**
1 scHiCDiff successfully detected DCIs in simulated data . . . 10
2 scHiCDiff effectively revealed cell type-specific DCIs . . . 13
3 scHiCDiff methods yielded consistent DCI detection results. . . . 14
4 Stable DCI detection results were produced by scHiCDiff . . . 15
5 scHiCDiff methods produced more reliable DCIs than the set-difference approach . . 16
6 scHiCDiff detection results were supported by bulk Hi-C differential analysis methods 18
7 DCIs detected by scHiCDiff were consistent with the dynamics of epigenetics features and gene expression . . . 19

Supplementary References **21**

Supplementary Methods

The scHiCDiff software tool identifies the changes of chromatin interactions in single-cell Hi-C (scHi-C) data through two main steps: the first step is data imputation and normalization (Section 1) and the second step is differential detection tests (Sections 2-3). In addition, the simulation settings are explained in Section 4 and real data pre-processing is outlined in Section 5.

1 Data imputation and normalization

Imputation has become a common pre-processing step in many scHi-C data analyses due to the extreme sparsity of contact matrices [1, 2]. The missing values in the contact matrix can arise from experimental limitations and dropout events and do not necessarily indicate the absence of interactions. To address this issue, we use a 2D Gaussian convolution step to infer the missing values. Given that the genome is linearly connected, we assume that the interaction partners of a particular bin are likely to be in proximity to its neighboring bins.

Specifically, we substitute the read count of each contact ($\dot{c}_{ij}$) in the contact map $\dot{C}^{n\times n}$ with the Gaussian weighted counts ($\tilde{c}_{ij}$) of all contacts in its genomic neighborhood, defining as follows:

$$\tilde{c}_{ij} = \sum_{k=\max(1,i-h)}^{\min(i+h,n)} \sum_{l=\max(1,j-h)}^{\min(j+h,n)} w_{kl}\dot{c}_{kl}$$

The filter F of size $(2h+1)\times(2h+1)$ is used to define the weights for neighboring bin pairs. The weight w_{kl} for a neighboring bin pair (k,l) is defined as $w_{kl} = \frac{G(k-i,l-j)}{\sum_{r=\max(1,i-h)}^{\min(i+h,n)} \sum_{s=\max(1,j-h)}^{\min(j+h,n)} G(r-i,s-j)}$. Here, $G(x,y) = \frac{1}{2\pi\sigma^2}e^{\frac{x^2+y^2}{2\sigma^2}}$ is the Gaussian kernel, and the default values for the window size h and the standard deviation of the Gaussian kernel σ are 1 and 0.5, respectively.

As with bulk Hi-C data, scHi-C data is prone to systematic biases related to the effective length, GC content, and mappability of chromatin fragment ends. To mitigate these biases, we normalize the data by scHiCNorm [3] with the negative binomial hurdle option, where the raw contact counts are assumed to follow a negative binomial hurdle distribution and fitted by a regression model with the three local bias features.

Consider m imputed scHi-C contact frequency matrices $\tilde{C}^k \in N^{n\times n}$, for $k = 1,\cdots,m$, where $\tilde{c}^k_{ij}$ is the interaction frequency between bins i and j in the k-th cell ($1 \leq i,j \leq n$, and $1 \leq k \leq m$). Here, we apply scHiCNorm [3] which assumes the observed contact frequency $\tilde{c}^k_{ij}$ in the scHi-C matrix follows a negative binomial hurdle model. After fitting a regression model per chromosome per cell with bias features being variables, the normalized contact frequency $\hat{c}^k_{ij}$ is calculated as the observed frequency $\tilde{c}^k_{ij}$ divided by the estimated mean of the regression model $\hat{\mu}^k_{ij}$.

Like bulk Hi-C data, the genomic distance effect, whereby genomic bin pairs that are proximal along the chromosome exhibit more Hi-C contacts than distal bin pairs, dominates every scHi-C matrix. In addition, the form of such distance effect also varies among different single cells. Therefore, we introduce a size factor to account for the genomic distance bias. Here, we enforce that the median normalized frequency for bin pairs at each given distance in each matrix to be the same. Specifically, for each distance $d \in \{0,\cdots,n-1\}$, the size factor $\hat{s}^k_d$ is calculated as

$$\hat{s}^k_d = \operatorname*{median}_{|i-j|=d} \hat{c}^k_{ij}.$$

Thus, the final normalized frequency between bins i and j in the k-th cell is given by

$$c_{ij}^k = \frac{\hat{c}_{ij}^k}{\hat{s}_{|i-j|}^k}.$$

2 Detecting differential chromatin interactions by non-parametric tests

After normalization, the m normalized scHi-C matrices $\{C^k \in N^{n\times n}, k = 1, \cdots, m\}$ are generated, where c_{ij}^k represents the normalized contact frequency between bins i and j in the k-th cell (for $1 \le i, j \le n$, and $1 \le k \le m$). Suppose each single-cell experiment is performed in one of two conditions $\mathbb{A} = \{condition\ 1, condition\ 2\}$. We denote by $\rho(k) \in \mathbb{A}$ as the condition corresponding to the k-th scHi-C matrix, for $1 \le k \le m$. For *condition* $A \in \mathbb{A}$, $A = 1$ or 2, we denote m_A as the number of scHi-C matrices belonging to *condition* A. In particular, it holds that $m_1 + m_2 = m$.

To detect the difference of contact frequencies for a specific bin pair between two conditions, it is equivalent to comparing the empirical distributions of contact frequencies in different conditions. Here, we consider two non-parametric methods: Kolmogorov–Smirnov test and Cramér-von Mises test. For both tests, the null hypothesis is that the two groups of cells are drawn from the same distribution. The premise of these non-parametric tests is that when the cells are drawn from two populations with the same distribution, the test should result in a high p-value; otherwise, if the two groups are drawn from different populations of cells, then the resulting p-value should be low.

2.1 Kolmogorov–Smirnov test

The Kolmogorov-Smirnov (KS) test [4] captures the maximum absolute difference (L1 norm) between the empirical cumulative distribution functions (ECDFs) of two populations. Specifically, for a bin pair (i, j), given that ECDFs of the normalized contact frequencies in the two conditions are $F_1(x)$ and $F_2(x)$, respectively, the KS test statistics is defined as

$$D_{m_1,m_2} = \max_x |F_1(x) - F_2(x)|.$$

When m_1 and m_2 are large enough, the null hypothesis that two samples are drawn from the same distribution is rejected at significant level α if

$$D_{m_1,m_2} > c(\alpha)\sqrt{\frac{1}{m_1} + \frac{1}{m_2}},$$

where $c(\alpha) = \sqrt{-ln(\frac{\alpha}{2}) \times \frac{1}{2}}$ by [5].

Thus, the p-value associated with the null hypothesis can be calculated as

$$p(D_{m_1,m_2}) = 2e^{\frac{2m_1m_2}{m_1+m_2}}D_{m_1,m_2}^2.$$

2.2 Cramér-von Mises test

The Cramér-von Mises (CVM) test [6] improves on the KS test using the full joint sample and compares two ECDFs by measuring the sum of the squared differences between them instead of

maximum distance. That is,

$$T = \frac{m_1 m_2}{m_1 + m_2} \int_{-\infty}^{\infty} (F_1(x) - F_2(x))^2 dF_{1+2}(x),$$

where $F_1(x)$ and $F_2(x)$ denote the ECDFs of normalized contact frequencies of bin pair (i, j) in the two conditions, respectively, and $F_{1+2}(x)$ is the ECDF of all normalized contact frequencies of bin pair (i, j) in both conditions.

Suppose that $(r_1, ...r_l, .., r_{m_1})$ and $(s_1, ..., s_t, ..., s_{m_2})$ represent the ranks of the contact frequencies in the two conditions, in the ordered pooled samples, then the CVM test statistic T could be rewritten as

$$T = \frac{U}{m_1 m_2 (m_1 + m_2)} - \frac{4 m_1 m_2 - 1}{6(m_1 + m_2),}$$

where

$$U = m_1 \sum_{l=1}^{m_1} (r_l - l)^2 + m_2 \sum_{t=1}^{m_2} (s_t - tk)^2.$$

Then the p-value associated with the null hypothesis that two samples are drawn from the same distribution can be computed as

$$p(T) = 1 - \frac{1}{\pi \sqrt{T}} \sum_{w=0}^{\infty} \frac{\Gamma(w + 0.5)}{\Gamma(0.5) w!} (4w + 1)^{0.5} e^{\frac{-(4w+1)^2}{16T}} K_{0.25} \frac{(4w + 1)^2}{16T},$$

where $\Gamma(z)$ is Euler's Gamma function, and $K_v(z)$ is a modified Bessel function of the second kind.

3 Detecting differential chromatin interactions by parametric models

While non-parametric tests enable the detection of DCIs in any scHi-C dataset without prior assumption on the data distribution, parametric models are more widely used in most comparative analyses by customizing more suitable models for specific cases. In particular, Poisson and Negative Binomial (NB) models have been commonly applied in bulk Hi-C data analyses [7, 8, 9, 10].

To evaluate the model fitting in scHi-C data, we attempt to fit scHi-C matrices of 280 diploid mouse embryonic stem cells (mESCs) at early-S stage in Nagano et al. [11] with both Poisson and NB models. According to the likelihood ratio tests on 185,056 bin pairs between Poisson and NB models, the majority (59.25%) indicate statistically significantly better fitting with the NB model than those with the Poisson model (Figure S1(b)(c)). Thus, we prefer to apply the NB model in scHi-C differential analysis.

The scHi-C data is extremely sparse (Figure S1(a)), and the excessive zeros in the contact matrices would lead to over-dispersion. However, NB model is not designed for dealing with such sparse observations, and cannot fully explain the excessive zeros. Therefore, we further adopt the zero-inflated Negative Binomial (ZINB) model to account for the excessive zeros and we find it fits the scHi-C data well (Figure S1(d)).

3.1 Negative Binomial model

In the NB model, we assume the contact frequency C_{ij} of bin pair (i, j) follows a negative binomial distribution with mean μ_{ij} and dispersion α_{ij}. Then the probability mass function (PMF) of C_{ij}

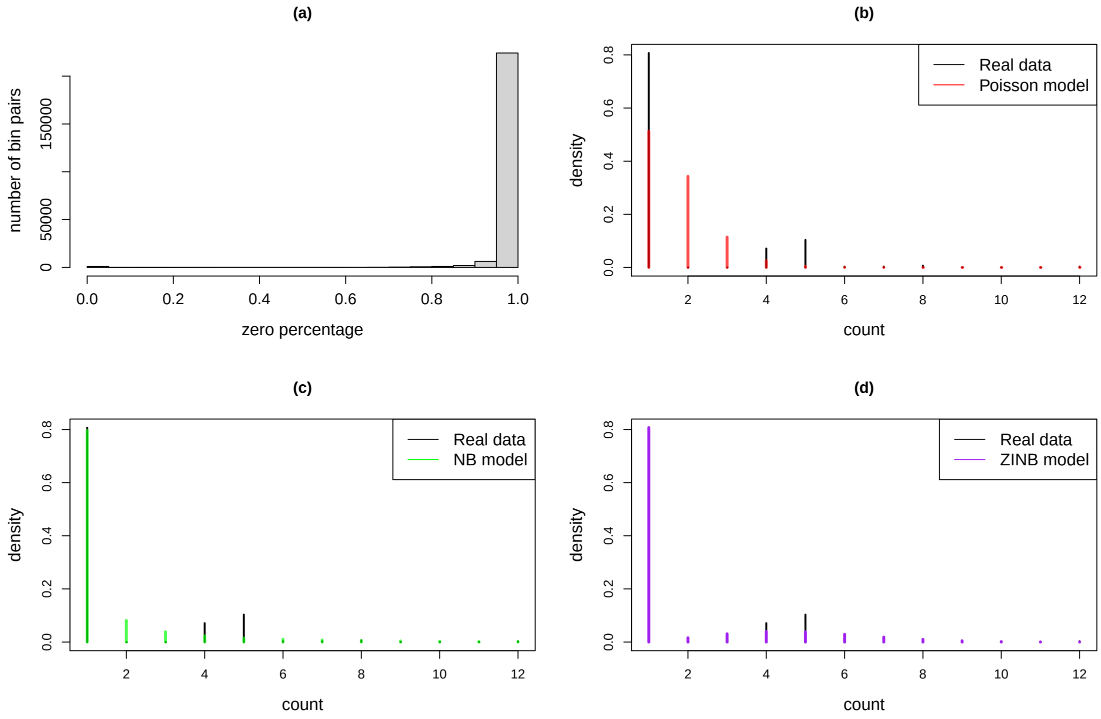

Figure S1: Summary of scHi-C dataset in diploid mESCs with resolution of 200 kb. (a). Histogram of zero percentages of contact frequencies for all bin pairs in a real scHi-C dataset [11]. (b)-(d). Fitting of parametric models for contact frequencies in a specific bin pair: black for real scHi-C data, and read, green, and purple panels show the density fitting of Poisson, NB, and ZINB models, respectively.

is written as

$$P(C_{ij}=c_{ij})=f(c_{ij})=\frac{\Gamma(c_{ij}+(\alpha_{ij})^{-1})}{c_{ij}!\Gamma((\alpha_{ij})^{-1})}\left(\frac{(\alpha_{ij})^{-1}}{\mu_{ij}+(\alpha_{ij})^{-1}}\right)^{(\alpha_{ij})^{-1}}\left(\frac{\mu_{ij}}{\mu_{ij}+(\alpha_{ij})^{-1}}\right)^{c_{ij}}.$$

When it comes to identifying differential interactions between two conditions, it is equivalent to testing the heterogeneity of two populations. Each population is characterized by a NB model; if any of the parameters of the two NB models has significant difference, the bin pair can be considered as differential.

Specifically, for bin pair (i,j), the difference of the two conditions are tested in the following three steps:

(1). Calculate the Maximum Likelihood Estimation (MLE) of the two NB populations' parameters $\hat{\Theta}_{ij,1}=\{\hat{\mu}_{ij,1},\hat{\alpha}_{ij,1},\hat{\mu}_{ij,2},\hat{\alpha}_{ij,2}\}$ with the Expectation-Maximization (EM) algorithm for each condition separately.

(2). Calculate the constrained MLE of the two NB populations' parameters $\hat{\Theta}_{ij,0}=\{\hat{\mu}_{ij,0},\hat{\alpha}_{ij,0}\}$ under the null hypothesis $H_0:\mu_{ij,1}=\mu_{ij,2},\alpha_{ij,1}=\alpha_{ij,2}$. It is equivalent to calculate the unconstrained MLE using the pooled data from two conditions.

(3). Hypothesis testing of H_0. Under the null hypothesis H_0, the likelihood-ratio test statistics χ^2_{LR} follows a χ^2_2 distribution. That is,

$$\chi^2_{LR}=-2\left[l(\hat{\Theta}_{ij,0}|m)-l(\hat{\Theta}_{ij,1}|m)\right]\sim\chi^2_2,$$

where $l(\Theta_{ij}|m)$ is the log-likelihood function. Then the hypothesis testing of H_0 is conducted using the χ^2_{LR} statistics.

3.2 Zero-inflated Negative Binomial model

The ZINB distribution is a mixture of constant zeros and a NB distribution with a mixing parameter p. That is, the zero counts of ZINB model come from two populations: a always-zero set and a NB distribution; the non-zero counts only come from the NB distribution. The PMF of ZINB distribution for contact frequency C_{ij} of bin pair (i,j) in a group of cells is defined as:

$$P(C_{ij}=c_{ij})=\begin{cases}p_{ij}+(1-p_{ij})f(0) & c_{ij}=0\\ (1-p_{ij})f(c_{ij}) & c_{ij}>0\end{cases}$$

where $f(z)$ is the density function of NB model with mean parameter μ_{ij} and dispersion parameter α_{ij}, and p_{ij} is the mixing parameter for the constant zeros.

When it comes to testing on the difference of the two ZINB populations, we perform similar likelihood-ratio testing steps as in the NB model:

(1). Calculate the MLE of the two ZINB populations' parameters $\hat{\Theta}_{ij,1}=\{\hat{p}_{ij,1},\hat{\mu}_{ij,1},\hat{\alpha}_{ij,1},\hat{p}_{ij,2},\hat{\mu}_{ij,2},\hat{\alpha}_{ij,2}\}$ with the EM algorithm for each condition separately.

(2). Calculate the constrained MLE of the two ZINB populations' parameters $\hat{\Theta}_{ij,0}=\{\hat{p}_{ij,0},\hat{\mu}_{ij,0},\hat{\alpha}_{ij,0}\}$ under the null hypothesis $H_0:p_{ij,1}=p_{ij,2},\mu_{ij,1}=\mu_{ij,2},\alpha_{ij,1}=\alpha_{ij,2}$. It is equivalent to calculate the unconstrained MLE using pooled data from two conditions.

(3). Hypothesis testing of H_0. Under the null hypothesis H_0, the likelihood-ratio test statistics χ^2_{LR} follows a χ^2_3 distribution. That is,

$$\chi^2_{LR} = -2\left[l(\hat{\Theta}_{ij,0}|m) - l(\hat{\Theta}_{ij,1}|m)\right] \sim \chi^2_3,$$

where $l(\Theta_{ij}|m)$ is the log-likelihood function. Then the hypothesis testing of H_0 is conducted using the χ^2_{LR} statistics.

Note that all non-parametric and parametric frameworks account for the multiple testing correction using the Benjamini-Hochberg procedure [12].

4 Simulation setting

To assess the performance of non-parametric and parametric methods in scHiCDiff, we conduct a series of comparisons on simulated datasets. First, we use the scHi-C dataset at G1 stage (280 cells) in diploid mESCs cultured with 2i from Nagano et al. [11] (GEO accession: GSE94489) as the base for the simulation.

Here, we outline a sequence of steps to generate simulation data as follows:

(1). Merge data from k-sampled single cells to create a pseudo-bulk data;
(2). Generate a pair of pseudo-bulk data: one representing the treatment condition with a pre-specified set of DCIs, and the other one representing the control condition which is identical to the original pseudo-bulk data;
(3). Simulate k single-cell data in both the treatment and control conditions, by downsampling from the corresponding pseudo-bulk data for each condition.

These steps enable the creation of simulated single-cell Hi-C data for both treatment and control conditions, facilitating controlled assessments of method performance. Specifically, in step (2), we use the pseudo-bulk Hi-C matrix that is merged from k single cells as a reference to simulate the contact frequencies in both the treatment matrix and the control matrix. Here, we randomly select a small subset (approximately 1%) of non-zero bin pairs whose interaction frequencies are above the average, and we designate these bin pairs as DCIs between the treatment and control groups. In the treatment matrix, the interaction frequencies of these pre-chosen bin pairs are increased by multiplying the original counts with a given fold change, while the contact counts in the control matrix remain the same as the original pseudo-bulk Hi-C.

Later in step (3), the contact frequencies at bin pair (i, j) in the single cell matrices are simulated from multinomial distribution that accounting for both the total contact frequencies in each cell as well as the corresponding contact frequencies of bin pair (i, j) in original scHi-C matrices. Here, we set the weights of the cell-wise and bin-pair-wise factors to 0.1 and 0.9, respectively, to ensure the sparsity and similarity of simulated scHi-C matrices compared to the original ones.

Next, we conduct a series of simulation experiments by varying the factors with respect to the following three aspects:

(1). fold change, i.e., the discrepancy degree in contact frequencies between two conditions. Here, we set the fold change to 2.5, 5, and 10;
(2). resolution of scHi-C matrices, from 50 kb to 1 Mb, which evaluates the sensitivity of our methods with respect to data sparsity level; and

(3). sample size k, i.e., the number of single cells in each condition, which is set ranging from 20 to 100.

At the default setting, we pre-define the fold change of DCIs to be 5 with the resolution of 200 kb and the sample size of 50 for each condition. Then, we change one of the three factors (fold change, resolution, or sample size) each time for comparison. For each simulation factor setting, we generate 20 iterations to evaluate the performance.

We further conduct Receiver Operating Characteristic (ROC) [13] and Precision Recall Curve (PRC) [14] analyses on simulated data to evaluate the performance of the methods in scHiCDiff by utilizing `PRROC` package in R. Specifically, the bin pairs with a fold change increase in the treatment matrix of step (2) are designated as the DCIs between the two conditions, while other bin pairs are considered non-DCIs. The true (false) positives are the bin pairs simulated to be (not to be) DCIs and reported as DCIs by scHiCDiff; while the false (true) negatives are the ones that are (not) DCIs in the simulation and are identified as non-DCIs by scHiCDiff.

5 Real data pre-processing

To assess the reliability of our methods on real data, we performed comparative analysis on the Flyamer et al. dataset (GEO accession: GSE80280) [15] and the Lee et al. dataset (GEO accession: GSE130711) [16]. For the Flyamer et al. dataset, it contains scHi-C data from mouse oocytes and zygotes with corresponding 98 and 88 single cells at 200-kb resolution. For quality control, we first rule out the cells with less than 5000 non-diagonal contacts, resulting in 143 remaining cells (89 oocyte cells and 54 zygote cells).The data are then imputed by Gaussian convolution and normalized using scHiCNorm [3] with genomic distance adjustment.

As to the Lee et al. dataset, we compare scHi-C data from human brain prefrontal cortex cell types, astrocytes (Astro) and endothelial cells(Endo), at 50-kb resolution. With the same data pre-processing and filtering procedures as previously described, all single cells (133 and 56) are retained from the original Astro and Endo cells in batch 190305. Due to the extreme sparsity and randomness of long-range interactions, we only utilize the contacts within the range of 0-10Mb for DCI analyses.

In addition to scHi-C data, ChIP-seq data in human Astro and Endo cells are also analyzed to evaluate the DCI detection results in the Lee et al. dataset [17]. The ChIP-seq datasets of CTCF and histone modifications (H3K27me3 and H3K27ac) are obtained from the ENCODE project [18].The ChIP-seq peak files are in narrowpeak BED format. The ChIP-seq peaks are aggregated into bins with 50kb and the bin-wise peak counts are normalized by the total number of peaks in each ChIP-seq dataset. The absolute mean differences of the normalized bin-wise peak counts are calculated between the two cell lines for the subsequent analyses.

Supplementary Notes

Supplementary Note 1: scHiCDiff successfully detected DCIs in simulated data

To validate the efficiency of our scHiCDiff methods, we evaluated their performance on three different aspects: fold change, resolution, and sample size. As shown in Figures S2 and S3, our results consistently indicated that the parametric models (NB and ZINB) outperformed the non-parametric tests (KS and CVM). One possible explanation for this difference in performance is that the non-parametric tests may not take into account the specific properties of scHi-C data.

Specifically, we assessed the accuracy of scHiCDiff methods on scHi-C matrices with various levels of fold change (2.5, 5, and 10) in the pre-defined DCIs between two conditions. As expected, scHiCDiff was able to detect the majority of the introduced differences with relatively low numbers of false positives, and the power of detecting DCIs increased dramatically as the fold change increased (Figures S2 and S3(a)-(c)). Moreover, we examined the influence of sample size on the detection of DCIs using different methods in scHiCDiff. In the case of non-parametric tests, there is no significant enhancement in detection performance as the sample size increases. However, for parametric models, the ROC curves demonstrated increased power in identifying DCIs with higher numbers of single cells per experimental condition (Figures S2 and S3(d)-(f)). This phenomenon is likely attributed to the fact that parametric models require a sufficient amount of data information to effectively conduct DCI detection. In addition, the ROC curves demonstrated the superiority of the ZINB model in improving the DCI detection power as it was specifically designed for handling the excessive sparsity problem in scHi-C data. Typically, Hi-C data at finer resolution have a higher proportion of zero contact frequencies (i.e., higher sparsity). As expected, we observed that the ZINB model performed better than other models at finer resolutions, with a more significant difference in the AUC values (Figure S2(g)-(i)). Collectively, these analyses demonstrated that parametric models in scHiCDiff can effectively detect DCIs in scHi-C data, and among them, the ZINB model produced the most accurate results in all simulation settings.

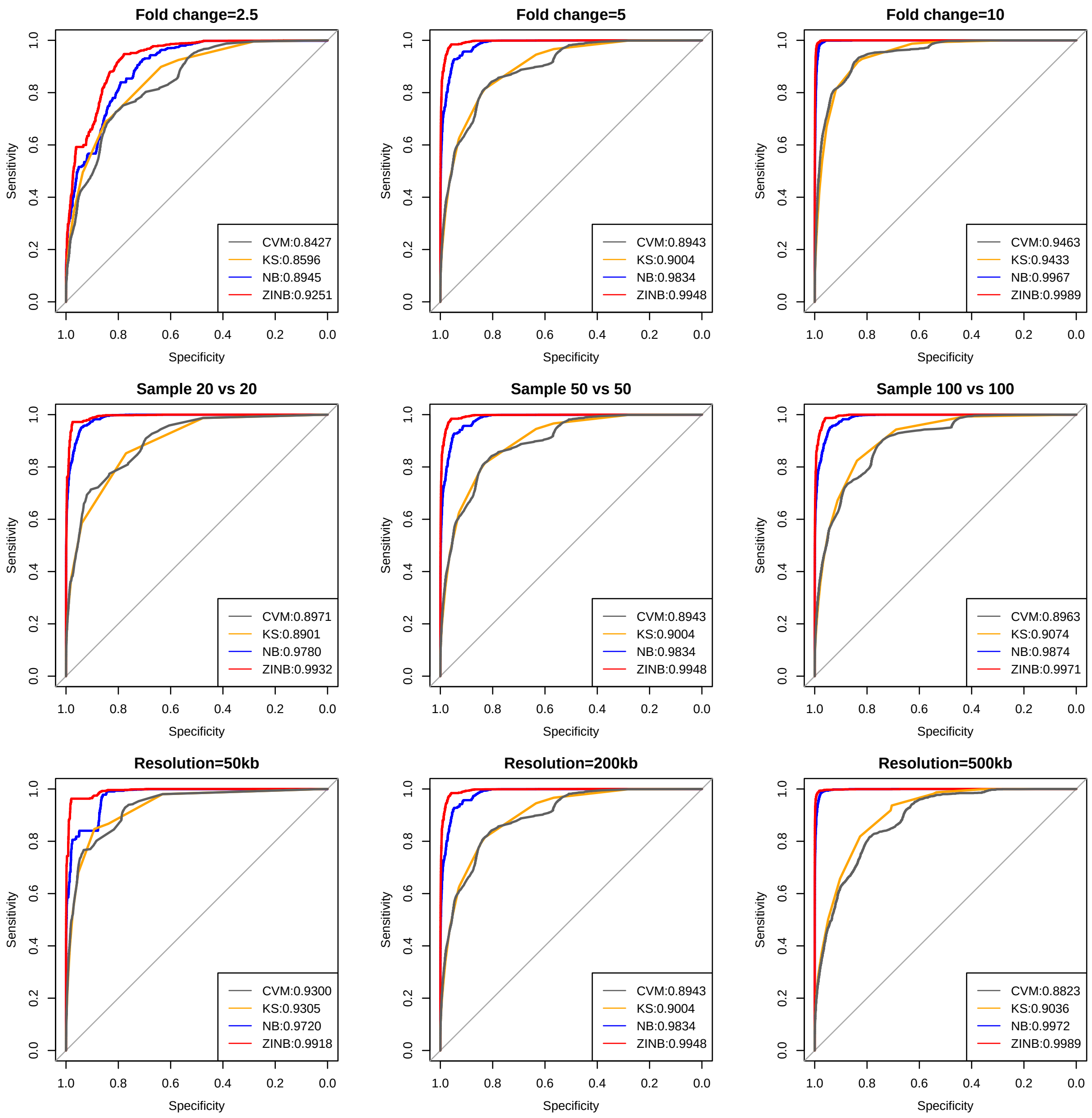

Figure S2: Performance of scHiCDiff methods on the simulated data. ROC curves of four differential analysis methods (NB, ZINB, KS, and CVM) in scHiCDiff are displayed in each subplot. The corresponding area under the PRC curve (AUC) values are listed in the bottom right corner. In the default setting ((b),(e),(h)), each set generates 20 simulations with fold change = 5, resolution = 200 kb, and sample size for each condition = 50. Then, one of the three factors is altered at each time: (a)-(c) fold change of 2.5, 5, and 10; (d)-(f) sample size of 20, 50, and 100; (g)-(i) resolutions of 50 kb, 200 kb, and 500 kb.

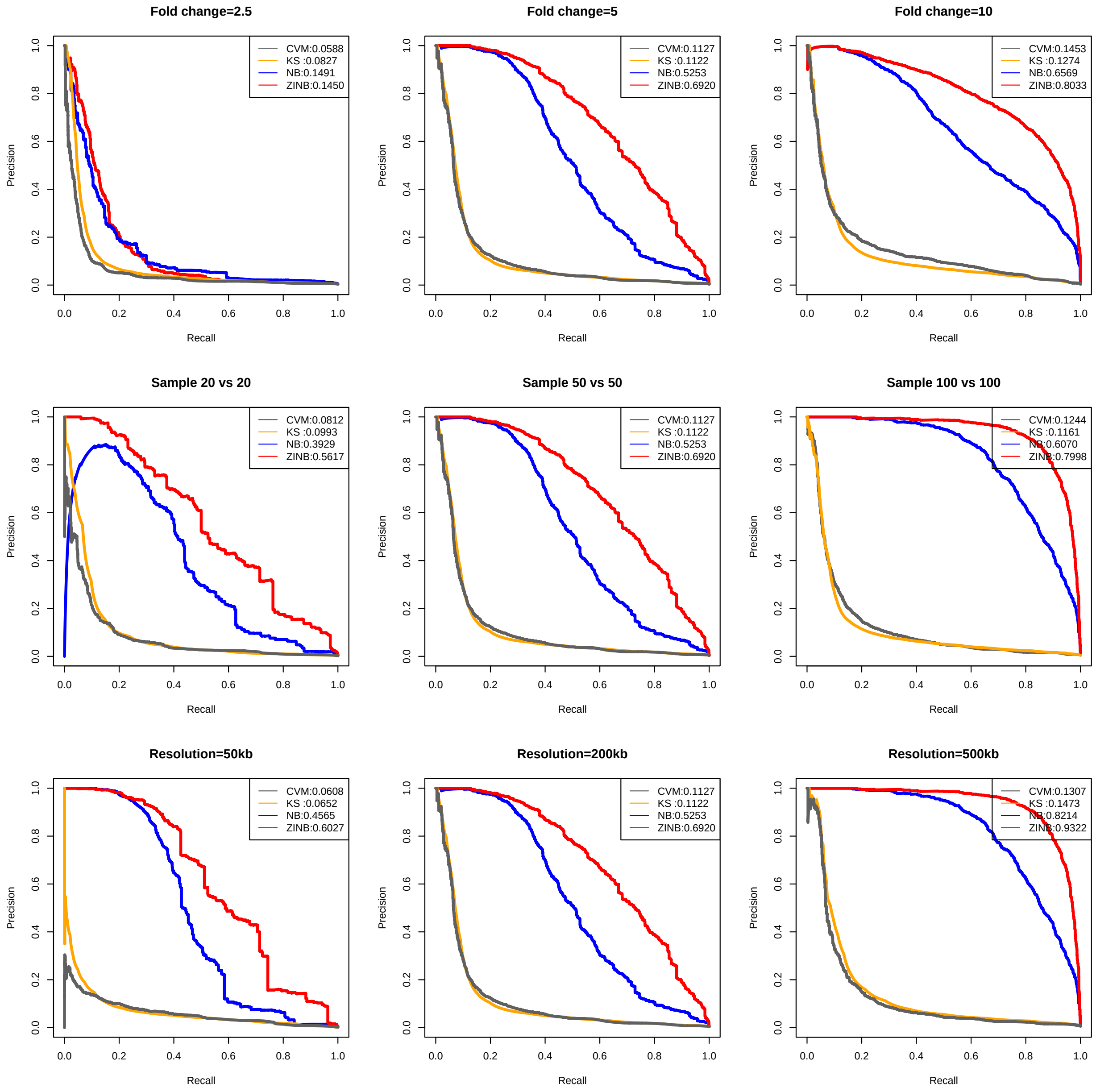

Figure S3: Performance of scHiCDiff methods on the simulated data. PRC curves of four differential analysis methods (NB, ZINB, KS, and CVM) in scHiCDiff are displayed in each subplot. The corresponding area under the PRC curve (AUC) values are listed in the top right corner. In the default setting ((b),(e),(h)), each set generates 20 simulations with fold change = 5, resolution = 200 kb, and sample size for each condition = 50. Then, one of the three factors is altered at each time: (a)-(c) fold change of 2.5, 5, and 10; (d)-(f) sample size of 20, 50, and 100; (g)-(i) resolutions of 50 kb, 200 kb, and 500 kb.

Supplementary Note 2: scHiCDiff effectively revealed cell type-specific DCIs

In addition to assessing scHiCDiff on simulated data, we further applied the four methods in scHiCDiff to two published scHi-C datasets: the Flyamer et al. dataset [15] and the Lee et al. dataset [16]. In the Flyamer et al. dataset, we conducted one comparison between oocyte cells of different conditions and the other comparison between oocyte and zygote cells at 200-kb resolution. We first sought to evaluate the performance of our algorithm between 24 active immature (non-surrounded nucleolus, NSN) oocytes and 62 transcriptionally inactive mature (surrounded-nucleolus, SN) oocytes. We hypothesized that a high degree of similarity in chromatin structures can be observed in cells of the same or similar type. As shown in Table S1, few DCIs were detected as expected. Specifically, no DCIs were identified by the two non-parametric tests KS, CVM, and the parametric model ZINB, while 293 bin pairs were detected as differential (with p-value < 0.05) across the whole genome (except for chrX) by the NB model. These results indicated that significant changes in chromatin interactions seldom occur between single cells of the same or similar type.

Next, we aimed to detect DCIs between oocyte and zygote cells. As illustrated in Table S1, 226,108 and 240,908 DCIs were detected by the non-parametric KS and CVM tests, respectively. As to our parametric models, it was shown that 243,571 and 191,394 DCIs were identified by the NB and ZINB models, respectively. These results confirmed that the scHiCDiff methods are capable of detecting DCIs between different cell types.

Table S1: Number of scHiCDiff-detected DCIs in oocyte and zygote scHi-C data.

	KS	CVM	NB	ZINB
Oocyte (NSN vs. SN)	0	0	293	0
Oocyte vs. Zygote	226,108	240,908	243,571	191,394

Besides the detection in the Flyamer et al. dataset, we also performed DCI analyses in the Lee et al. dataset. Similarly, we conducted one comparison of Endo cells between two batches (190305 and 190315) and another comparison between Astro and Endo cells from the same batch (190305) at 50-kb resolution. Table S2 summarizes the number of DCIs detected by scHiCDiff. Overall, all four methods in scHiCDiff efficiently captured substantively more DCIs between different cell types than between batches, demonstrating that scHiCDiff can effectively detect cell type-specific DCIs in scHi-C data.

Table S2: Number of scHiCDiff-detected DCIs in Astro and Endo scHi-C data.

	KS	CVM	NB	ZINB
Endo (batch 190305 vs. 190315)	0	0	253	235
Astro vs. Endo	123,709	87,619	11,392	1,593

Supplementary Note 3: scHiCDiff methods yielded consistent DCI detection results.

Although scHiCDiff successfully identified many DCIs between different cell types, the number of detected DCIs varied from one model to another. To further investigate the detection results, we conducted six pairwise consistency comparisons among four models in scHiCDiff. Here, we measured the consistent rate for every model pair, which was the number of bin pairs simultaneously appearing in the top 1% smallest p-value lists of both models over the number of bin pairs with the top 1% p-values.

Table S3 illustrates consistent detection results of DCIs among different model comparisons. Specifically, for DCIs between oocyte and zygote cells in the Flyamer et al. dataset at 200-kb resolution, the consistent rates of the top 1% list among method comparisons were relatively high. The concordant rates between the parametric models (NB versus ZINB) and between the non-parametric tests (KS versus CVM) were extremely high (over 80%). Due to the sparsity of the Lee et al. data at 50-kb resolution, the consistency performance of the DCI detection results between Astro and Endo cells was generally lower than the ones for oocyte versus zygote comparison. However, similar detection results appeared between the KS and CVM tests; the concordant rate between the two parametric models was over 90% in Astro versus Endo comparison. These observations of high consistent rate of detected DCIs could be explained by 1) the KS and CVM tests originated from the same idea but were measured with different distance norms in practice; 2) both parametric models NB and ZINB took the mean and dispersion of interaction counts in two cell types into consideration for differential testing. Overall, these results indicated that the four scHiCDiff methods produced largely consistent DCI detection results.

Table S3: Average proportions of commonly detected DCIs between model pairs.

	Oocyte vs. Zygote	Astro vs. Endo
KS vs. CVM	0.8767	0.7539
KS vs. NB	0.6739	0.3432
KS vs. ZINB	0.7645	0.3244
CVM vs. NB	0.7316	0.4471
CVM vs. ZINB	0.8351	0.4165
NB vs. ZINB	0.8204	0.9167

Supplementary Note 4: Stable DCI detection results were produced by scHiCDiff

Since the DCIs are inherently associated with the experimental conditions of study, they are likely to be largely stable between conditions. To investigate the stability of scHiCDiff in DCI detection between conditions, we randomly selected a partial set of cells for testing. Considering that the number of qualified cells in the Flaymer et al. dataset was 89 and 54 for oocytes and zygotes, respectively, and the simulation results indicated that the detection results are more reliable when the sample size for each condition is great than 50, we retained all 54 zygote cells and randomly chose 50 and 75 of 89 cells from oocytes for the stability test. Similar to the previous consistency test, we compared the DCI detection results from randomly-selected test datasets with the results from the original full dataset with p-value < 0.05.

Table S4 shows that all four detection methods in scHiCDiff produced highly consistent results between the randomly-sampled test datasets and the original full dataset, with a concordance rate of over 80%. Furthermore, as the number of randomly sampled oocyte cells increased from 50 to 75, the concordance with the detection results from the original full dataset also increased. Surprisingly, no significant improvement in the performance of the ZINB model was observed with increasing sample size, but its consistency was still the highest among the four detection methods. These findings suggest that our ZINB model can effectively identify significant DCIs between different cell types in a steady and reliable manner.

Table S4: Numbers and proportions of commonly detected DCIs for detection stability verification.

	Oocyte (50) vs. Zygote (54)		Oocyte (75) vs. Zygote (54)	
	# of DCIs	Consistent rate	# of DCIs	Consistent rate
KS	228,760	87.17%	232,986	90.24%
CVM	236,327	88.84%	243,587	91.04%
NB	210,449	95.68%	239,118	96.21%
ZINB	158,682	96.79%	187,942	96.38%

Supplementary Note 5: scHiCDiff methods produced more reliable DCIs than the set-difference approach

To further evaluate the effectiveness of scHiCDiff methods, we compared them to a simple set-difference approach that calculates the difference of significant chromatin interactions between two conditions. Specifically, we utilized SnapHiC [19], a recent method for identifying chromatin loops from scHi-C data, to detect significant chromatin interactions for each condition individually. We then computed the set differences of significant contacts between the two conditions and compared them with the results obtained from scHiCDiff.

We conducted this comparison using both simulated data and biological replicates in real scHi-C data. For the simulated data, we analyzed two specific settings: (1) a fold change of 5, sample size of 50 for each condition, and a resolution of 50 kb, and (2) a fold change of 5, sample size of 50 for each condition, and a resolution of 200 kb. The first setting was chosen as it is the default in our simulation, while the second setting was selected based on the reliable detection results obtained from SnapHiC with a fine resolution. Our assessment of precision, recall, and accuracy using a p-value threshold of 0.05 demonstrated that scHiCDiff methods outperformed the simple set-difference approach of calculating differences between significant interactions (Figure S4).

We also examined the significant interactions between biological replicates of real scHi-C data. Although minimal differences are expected between replicates of the same cell types, we observed 10,765 different significant interactions between the NSN and SN conditions in the 200-kb oocyte data, and 65,653 ones in the 50-kb Endo replicates between two batches (190305 and 190315). Notably, these numbers considerably exceeded the number of DCIs identified by scHiCDiff (Tables S1 and S2), highlighting the reliability of scHiCDiff in detecting differential interactions between two conditions.

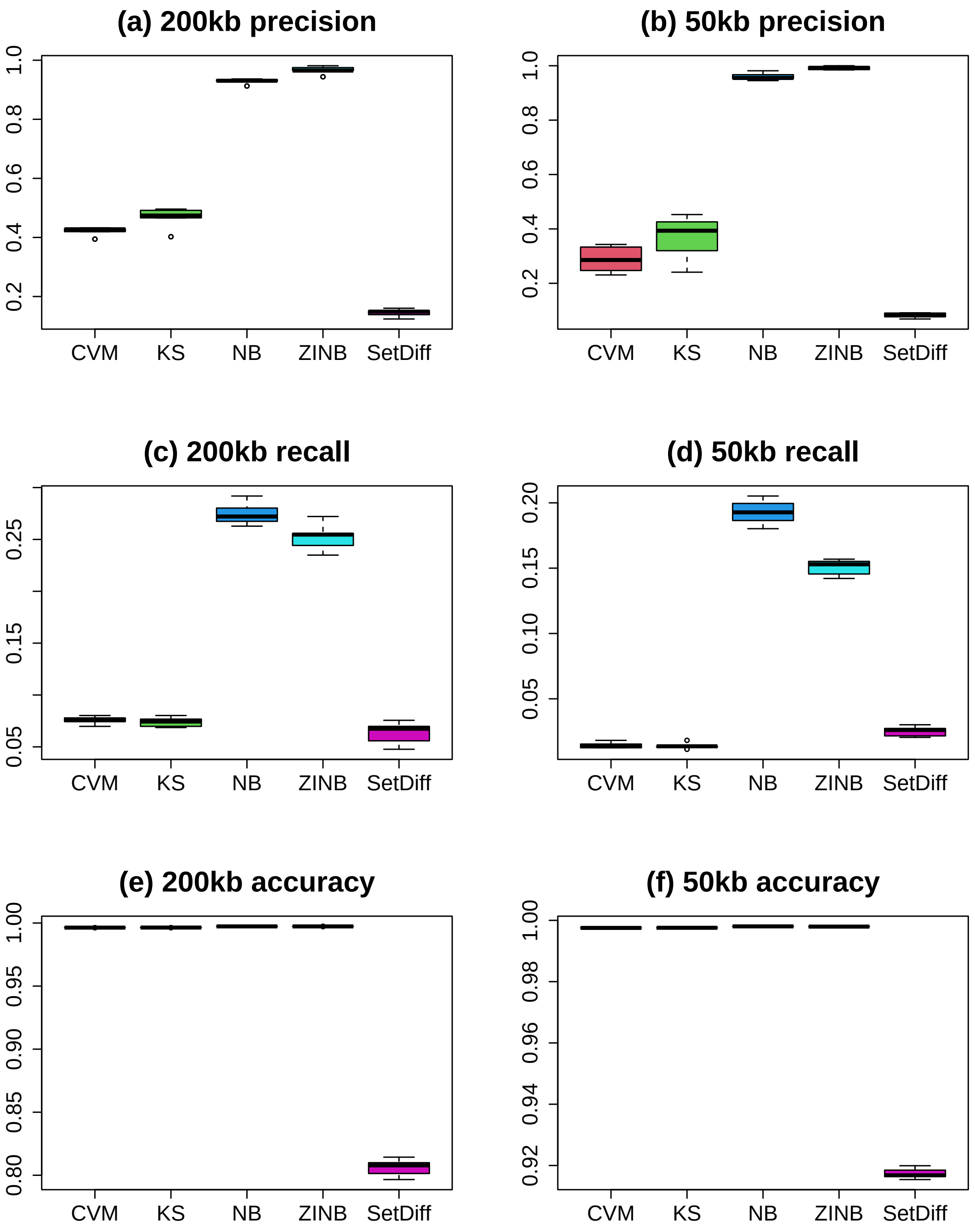

Figure S4: Boxplots of precision, recall, and accuracy for the results from scHiCDiff models and the set-difference approach. (a),(c), and (e): simulation setting of fold change = 5, sample size = 50, and resolution = 200 kb. (b),(d), and (f): simulation setting of fold change = 5, sample size = 50, and resolution = 50 kb.

Supplementary Note 6: scHiCDiff detection results were supported by bulk Hi-C differential analysis methods

To evaluate the performance of scHiCDiff methods, we first compared them with existing methods for detecting differential chromatin interactions (DCIs) in bulk Hi-C data. Since there is no ground truth for DCIs in real scHi-C data across different cell types, we used two established methods for generating reference lists of DCIs in bulk Hi-C: diffHic [8], which utilizes a generalized linear model (negative binomial regression) from edgeR to identify DCIs, and FIND [7], which considers the dependency of adjacent loci at finer resolutions and uses a spatial Poisson process model to identify DCIs that show significant changes in interaction frequencies of both themselves and their neighboring bins.

For the Lee et al. dataset, due to the lack of corresponding bulk Hi-C data, we merged scHi-C data in each batch of Astro and Endo cells separately as pseudo-bulk matrices for the detection of differential interactions. We applied diffHic and FIND methods to the Astro and Endo pseudo-bulk data with two replicates for each cell type, and identified 140,417 and 454,025 differential interactions, respectively. Results in Table S5 showed that the ZINB model detected 71.12% of DCIs found by the FIND method, followed by the NB model with 68.44%, while the concordant proportions for both non-parametric tests (CVM and KS) were lower at 39.27% and 42.28%, respectively. Moreover, the parametric models showed better consistency with diffHic differential results than non-parametric models, indicating that parametric models performed better in terms of agreement with bulk Hi-C DCIs.

Previous bulk Hi-C comparative studies have shown that the majority of the chromatin interaction changes strongly correlated to topologically associated domains (TADs) [20, 21, 22]. Additionally, scHi-C studies have revealed that variable contact clusters in single cells averaged into population TADs when pooled together, and the locations of detected TAD borders were generally conserved in the pools of single-cell data from different cell cycles [11, 15]. Thus, we called TADs in the pooled pseudo-bulk matrices of Astro and Endo cells in Lee et al. dataset using HiCseg [23] and then identified differentially interacting genomic regions at TAD-level between the pooled Astro and Endo data using the DiffGR method [24]. We found that the consistent rates between DCIs detected by parametric and differential regions identified by DiffGR were higher than those given by non-parametric tests, with over 60% of DCIs detected by the ZINB model located within DiffGR-identified differential genomic regions between the two cell types.

Collectively, these results indicated that the ZINB model in scHiCDiff can reliably detect DCIs at the single-cell level, and that the DCI detection results were strongly supported by changes in interactions and TAD-level chromatin organizations at the bulk level. Based on the superior performance and high consistency of the ZINB model in considering excessive zeros, we utilized the results obtained from the ZINB model for subsequent analysis.

Table S5: Agreements between scHiCDiff and bulk Hi-C differential methods.

	FIND	diffHic	DiffGR
CVM	0.3927	0.1810	0.4698
KS	0.4228	0.2164	0.4783
NB	0.6844	0.3050	0.5364
ZINB	0.7112	0.4363	0.6108

Supplementary Note 7: DCIs detected by scHiCDiff were consistent with the dynamics of epigenetics features and gene expression

As there is no ground truth of DCIs available in real data, we sought to assess the performance of scHiCDiff by investigating the association between the changes of 1D epigenomic features and 3D chromatin interaction regions. As CTCF is a master controller of the chromatin architecture and plays important roles in gene regulation [20], we expect that DCIs are more likely to be located in the neighborhoods of CTCF differential binding sites. In addition, we also examined the ChIP-seq datasets of other histone modifications (H3K27ac and H3K27me3). Specifically, we compared the differential ChIP-seq peaks at the bin sites appearing within or without detected DCIs. On each chromosome, we evaluated whether the mean absolute differences of the ChIP-seq signal at the bins appearing within detected DCIs by the ZINB model were significantly higher (with a significant level of 0.1) than those appearing without detected DCIs.

We performed 22 chromosome-wide tests (excluding ChrX) for each scHiCDiff model and each ChIP-seq data between Astro and Endo cells. As shown in Table S6, 14 out of 22 tests showed higher differences of CTCF peaks at bins appearing in DCIs detected by ZINB, and half of them showed significantly higher differences. In addition, similar or higher agreements between scHiCDiff-detected DCIs and ChIP-seq data were also observed for H3K27ac and H3K27me3 (Table S6). Taken together, these results indicated that scHiCDiff-detected DCIs were highly concordant with differential bindings of transcription factors and histone modifications related to chromatin remodeling and regulation.

Table S6: Agreements between scHiCDiff-detected DCIs and ChIP-seq data.

	higher	significantly higher
CTCF	14	11
H3K27ac	13	11
H3K27me3	15	12

For each ChIP-seq data, a total of 22 chromosome-wide tests were conducted. The number of chromosomes (out of 22), whose mean absolute differential ChIP-seq peaks at the bins appearing in DCIs detected by ZINB were higher / significantly higher (based on t-test with p-value < 0.1) than those not appearing in DCIs, were recorded.

To further explore the potential functional roles of the changes in chromatin interactions on gene expression regulation, we performed Gene Ontology (GO) enrichment analysis on the genes located within highly differential loci. Specifically, we selected 2139 influenced bins that appeared in DCIs detected by the ZINB model between Astro and Endo cells, and then performed GO analysis on 1120 genes located within these loci using DAVID [25].

The functional analysis of the 1120 genes showed a high enrichment for cellular component GO terms of postsynaptic density and its membrane component, neuron projection, presynaptic membrane, and glutamatergic synapse, which are all related to the nervous system (Table S7). These findings were consistent with the neurologic nature of Astrocytes.

Table S7: Functional enrichment of genes located within the DCI sites

GO Term	FDR
GO:0014069 Postsynaptic density	2.6E-3
GO:0043005 Neuron projection	3.6E-3
GO:0042734 Presynaptic membrane	6.1E-3
GO:0098839 Postsynaptic density membrane	9.7E-3
GO:0098978 Glutamatergic synapse	1.3E-2

GO enrichment analysis was performed on the 1120 genes within the genomic bins where DCIs were located.

Supplementary References

[1] Tianming Zhou, Ruochi Zhang, and Jian Ma. The 3d genome structure of single cells. *Annual review of biomedical data science*, 4:21–41, 2021.

[2] Aleksandra A Galitsyna and Mikhail S Gelfand. Single-cell hi-c data analysis: safety in numbers. *Briefings in bioinformatics*, 22(6):bbab316, 2021.

[3] Tong Liu and Zheng Wang. schicnorm: a software package to eliminate systematic biases in single-cell hi-c data. *Bioinformatics*, 34(6):1046–1047, 2018.

[4] William Jay Conover. *Practical nonparametric statistics*, volume 350. john wiley & sons, 1999.

[5] Donald E Knuth. *Art of computer programming, volume 2: Seminumerical algorithms*. Addison-Wesley Professional, 2014.

[6] Theodore W Anderson. On the distribution of the two-sample cramer-von mises criterion. *The Annals of Mathematical Statistics*, pages 1148–1159, 1962.

[7] Mohamed Nadhir Djekidel, Yang Chen, and Michael Q Zhang. Find: differential chromatin interactions detection using a spatial poisson process. *Genome research*, 28(3):412–422, 2018.

[8] Aaron TL Lun and Gordon K Smyth. diffhic: a bioconductor package to detect differential genomic interactions in hi-c data. *BMC bioinformatics*, 16(1):258, 2015.

[9] Ming Hu, Ke Deng, Siddarth Selvaraj, Zhaohui Qin, Bing Ren, and Jun S Liu. Hicnorm: removing biases in hi-c data via poisson regression. *Bioinformatics*, 28(23):3131–3133, 2012.

[10] Kate B Cook, Borislav H Hristov, Karine G Le Roch, Jean Philippe Vert, and William Stafford Noble. Measuring significant changes in chromatin conformation with accost. *Nucleic acids research*, 48(5):2303–2311, 2020.

[11] Takashi Nagano, Yaniv Lubling, Csilla Várnai, Carmel Dudley, Wing Leung, Yael Baran, Netta Mendelson Cohen, Steven Wingett, Peter Fraser, and Amos Tanay. Cell-cycle dynamics of chromosomal organization at single-cell resolution. *Nature*, 547 (7661):61–67, 2017.

[12] Yoav Benjamini and Yosef Hochberg. Controlling the false discovery rate: a practical and powerful approach to multiple testing. *Journal of the Royal statistical society: series B (Methodological)*, 57(1):289–300, 1995.

[13] Tom Fawcett. An introduction to roc analysis. *Pattern recognition letters*, 27(8):861–874, 2006.

[14] J. Davis and M. Goadrich. The relationship between precision-recall and roc curves. In *Proceedings of the 23rd international conference on Machine learning*, pages 233–240, 2006.

[15] Ilya M Flyamer, Johanna Gassler, Maxim Imakaev, Hugo B Brandão, Sergey V Ulianov, Nezar Abdennur, Sergey V Razin, Leonid A Mirny, and Kikuë Tachibana-Konwalski. Single-nucleus hi-c reveals unique chromatin reorganization at oocyte-to-zygote transition. *Nature*, 544(7648):110–114, 2017.

[16] Dong-Sung Lee, Chongyuan Luo, Jingtian Zhou, Sahaana Chandran, Angeline Rivkin, Anna Bartlett, Joseph R Nery, Conor Fitzpatrick, Carolyn O'Connor, Jesse R Dixon, et al. Simultaneous profiling of 3d genome structure and dna methylation in single human cells. *Nature methods*, 16(10):999–1006, 2019.

[17] Hyeon-Jin Kim, Galip Gürkan Yardımcı, Giancarlo Bonora, Vijay Ramani, Jie Liu, Ruolan Qiu, Choli Lee, Jennifer Hesson, Carol B Ware, Jay Shendure, et al. Capturing cell type-specific chromatin compartment patterns by applying topic modeling to single-cell hi-c data. *PLoS Computational Biology*, 16(9):e1008173, 2020.

[18] ENCODE Project Consortium et al. The encode (encyclopedia of dna elements) project. *Science*, 306(5696):636–640, 2004.

[19] Miao Yu, Armen Abnousi, Yanxiao Zhang, Guoqiang Li, Lindsay Lee, Ziyin Chen, Rongxin Fang, Taylor M Lagler, Yuchen Yang, Jia Wen, et al. Snaphic: a computational pipeline to identify chromatin loops from single-cell hi-c data. *Nature methods*, 18(9): 1056–1059, 2021.

[20] Suhas SP Rao, Miriam H Huntley, Neva C Durand, Elena K Stamenova, Ivan D Bochkov, James T Robinson, Adrian L Sanborn, Ido Machol, Arina D Omer, Eric S Lander, et al. A 3d map of the human genome at kilobase resolution reveals principles of chromatin looping. *Cell*, 159(7):1665–1680, 2014.

[21] Jesse R Dixon, Inkyung Jung, Siddarth Selvaraj, Yin Shen, Jessica E Antosiewicz-Bourget, Ah Young Lee, Zhen Ye, Audrey Kim, Nisha Rajagopal, Wei Xie, et al. Chromatin architecture reorganization during stem cell differentiation. *Nature*, 518(7539): 331, 2015.

[22] Emily M Smith, Bryan R Lajoie, Gaurav Jain, and Job Dekker. Invariant tad boundaries constrain cell-type-specific looping interactions between promoters and distal elements around the cftr locus. *The American Journal of Human Genetics*, 98(1): 185–201, 2016.

[23] Celine Lévy-Leduc, Maud Delattre, Tristan Mary-Huard, and Stephane Robin. Two-dimensional segmentation for analyzing hi-c data. *Bioinformatics*, 30(17):i386–i392, 2014.

[24] Huiling Liu and Wenxiu Ma. Diffgr: Detecting differentially interacting genomic regions from hi-c contact maps. *bioRxiv*, 2020.

[25] Brad T Sherman, Richard A Lempicki, et al. Systematic and integrative analysis of large gene lists using david bioinformatics resources. *Nature protocols*, 4(1):44, 2009.
